# Supplementary material for: FIERY1 promotes microRNA accumulation by suppressing rRNA-derived small interfering RNAs in Arabidopsis
Source: Nat Commun. 2019 Sep 27;10:4424. doi: 10.1038/s41467-019-12379-z (PMC6765019; doi:10.1038/s41467-019-12379-z)
Supplement: Supplementary file 1 — Supplementary Information [file 41467_2019_12379_MOESM1_ESM.pdf]

Supplementary Table 1 Reads from sRNA-seq of *fry1* mutants and WT

	WT		*fry1-6*	
	1	2	1	2
Raw reads	23,303,045	18,752,487	15,854,975	13,763,566
Input reads	15,971,526	13,008,424	11,394,005	10,656,534
Unique mappers	3,482,516	2,639,103	1,631,593	1,607,459
Multi mappers	8,600,644	6,590,965	6,517,654	6,074,585
Unique mappers / input reads	21.80%	20.29%	14.32%	15.08%
Multi mappers / input reads	53.85%	50.67%	57.20%	57.00%
rRNA reads	3,399,389	2,493,744	2,887,144	2,581,968
rRNA/mapped reads	28.13%	27.02%	35.43%	33.61%
miRNA reads	578,344	370,874	197,478	201,095
miRNA/mapped reads	4.79%	4.02%	2.42%	2.62%

	fry1-8		*fry1-6 rdr6-11*	
	1	2	1	2
Raw reads	20,554,719	20,517,702	23042816	22703168
Input reads	13,795,730	14,774,170	16328665	16219281
Unique mappers	2,276,682	2,620,765	2948815	2939182
Multi mappers	7,768,896	8,506,505	7761040	7668080
Unique mappers / input reads	16.50%	17.74%	18.06%	18.12%
Multi mappers / input reads	56.31%	57.58%	47.53%	47.28%
rRNA reads	3,356,749	3,355,398	3,294,888	3300813
rRNA/mapped reads	33.42%	30.15%	30.77%	31.12%
miRNA reads	279,452	303,528	308403	298709
miRNA/mapped reads	2.78%	2.73%	2.88%	2.82%

Supplementary Figure 1

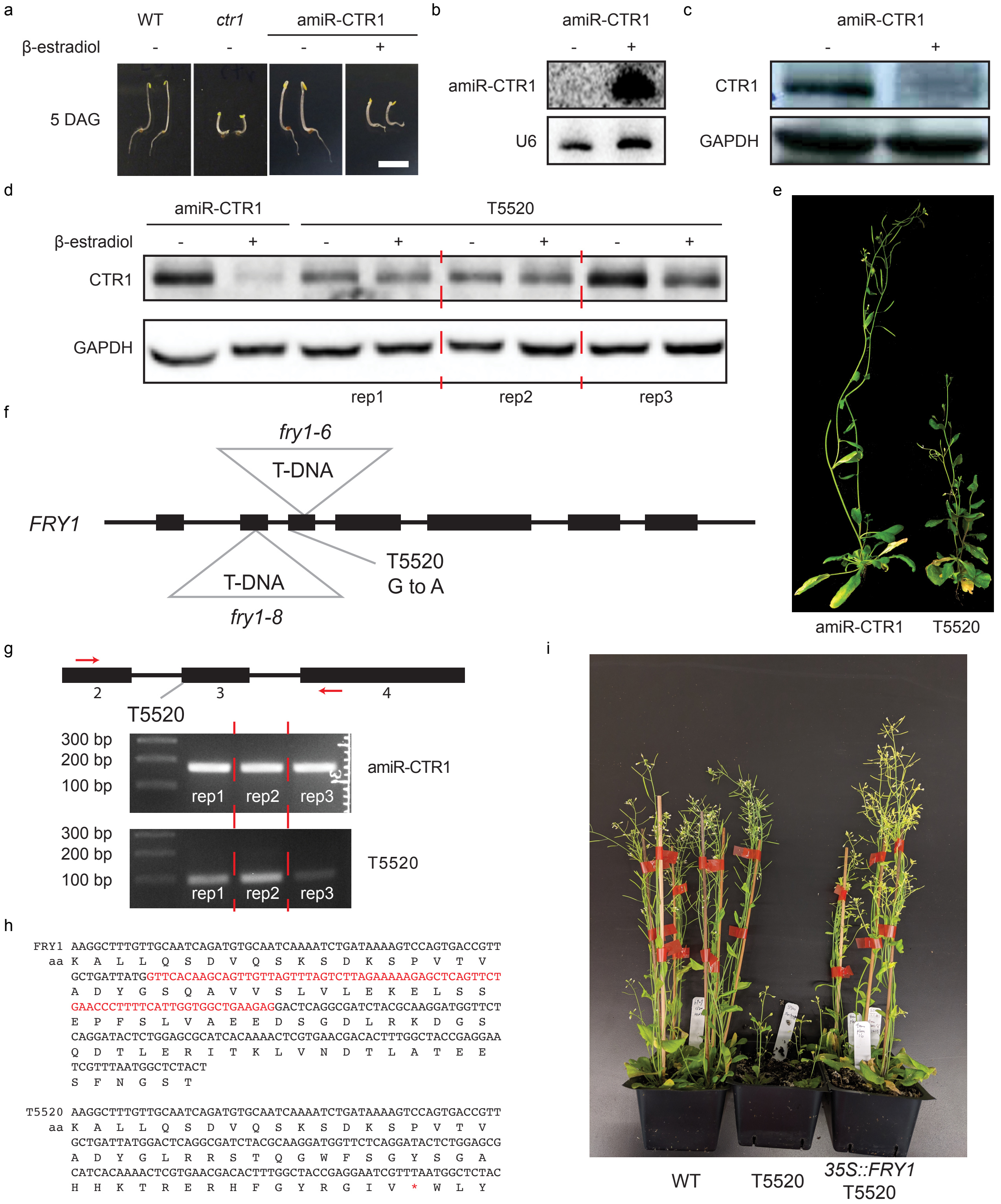

Supplementary Fig. 1 T5520 is a null allele of *FRY1*

a-**c**, amiR-CTR1 represses *CTR1* expression. Upon β-estradiol induction, amiR-CTR1 plants resemble a *ctr1* mutant (**a**). The accumulation of amiR-CTR1 upon β-estradiol induction is shown by an RNA gel blot assay (**b**). CTR1 protein levels decrease upon β-estradiol induction as determined by western blotting (**c**). Scale bar = 5 mm. **d**,**e**, The T5520 mutation suppresses amiR-CTR1. The levels of CTR1 protein are not suppressed by β-estradiol in T5520 as drastically as in amiR-CTR1 (**d**), and the T5520 plant is smaller and late-flowering (**e**). **f**-**h**, Identification of T5520. The mutation is at the acceptor site of the second intron of the *FRY1* gene. Two T-DNA lines used in this study are also show (**f**). To determine the consequence of the point mutation in T5520 on *FRY1* gene expression, a fragment flanking the mutation was amplified by RT-PCR. Three biological replicates show consistently shorter bands in T5520 compared to those in WT (**g**). The mutation caused an exon-skipping event (the red sequence indicating the skipped exon 3) according to Sanger sequencing and resulted in a pre-mature stop codon (the red asterisk) (**h**). **i**, Restoration of the T5520 phenotypes by *35S::FRY1*. Source data are provided as a Source Data file.

Supplementary Figure 2

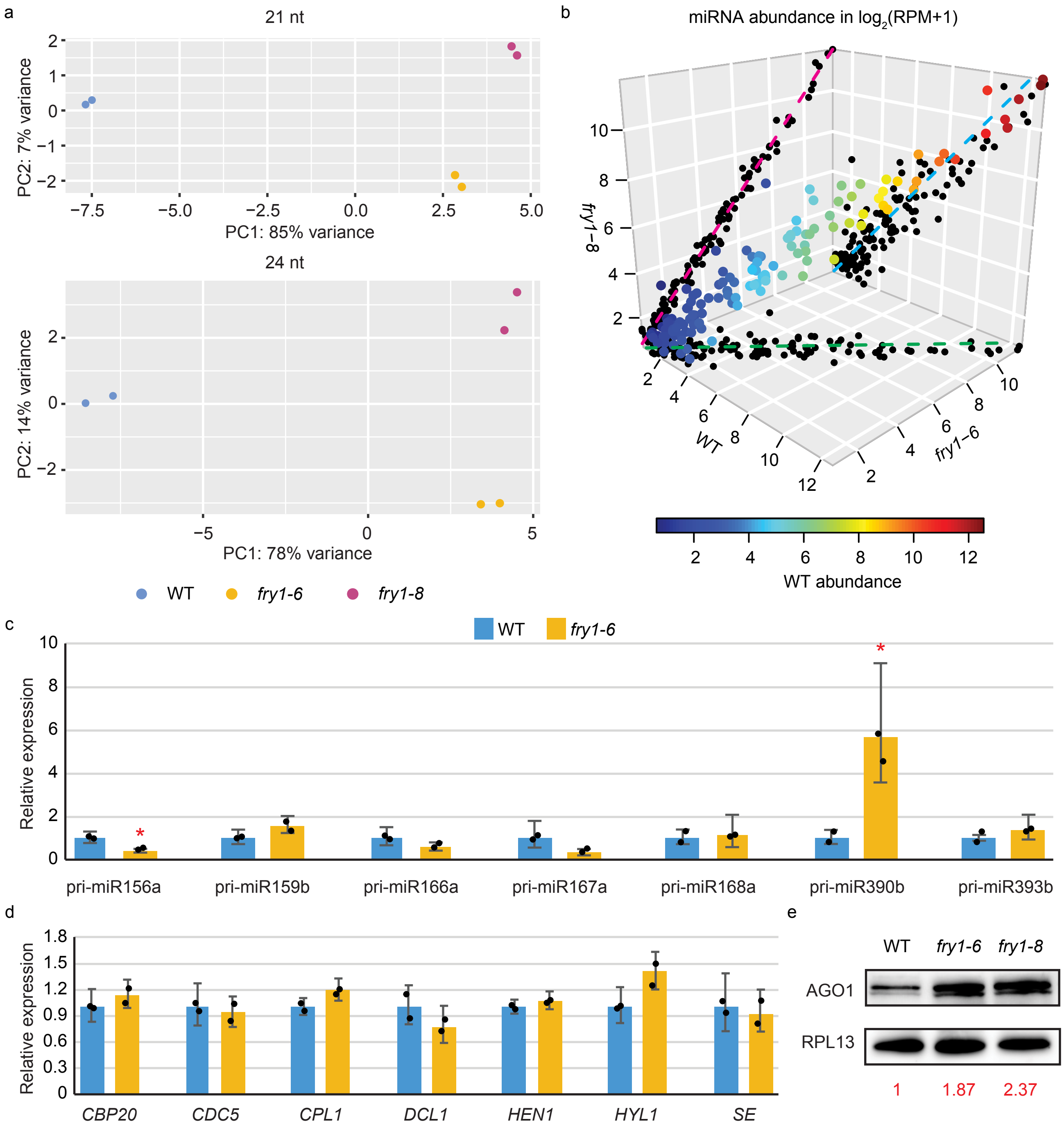

Supplementary Fig. 2 Reproducibility of sRNA-seq from WT, *fry1-6*, and *fry1-8*

a, Principal Component Analysis for 21-nt and 24-nt bins across WT, *fry1-6* and *fry1-8*. In addition to the good reproducibility of biological replicates in each genotype, the two *fry1* mutants are clustered and separated from WT. **b**, 3D plots for miRNA abundance in WT and *fry1* mutants. The coordinates of each dot represent the abundance of a detected miRNA in all three genotypes in log_2(RPM), and the color represents the abundance in WT. Three pairwise comparisons were shown as projections of dots (black dots). In *fry1-6* vs. WT and *fry1-8* vs. WT, the majority of dots align near the diagonal but are slightly closer to the WT axis WT, suggesting that many miRNAs show reduced levels in *fry1* mutants. **c**,**d**, Levels of pri/pre-miRNAs and mRNAs from genes involved miRNA biogenesis in *fry1-6*. Scale bars were defined by the SEM calculated from 2 biological replicates. P values were calculated by a two-tailed Student's t test. One asterisk, P value < 0.05; two asterisks, P value < 0.01. **e**, AGO1 levels as determined by western blotting. AGO1 levels increase in both *fry1* mutants. RPL13 was used as a loading control to estimate the relative AGO1 abundance (as indicated by the numbers below the blots) among the three genotypes. Source data are provided as a Source Data file.

Supplementary Figure 3

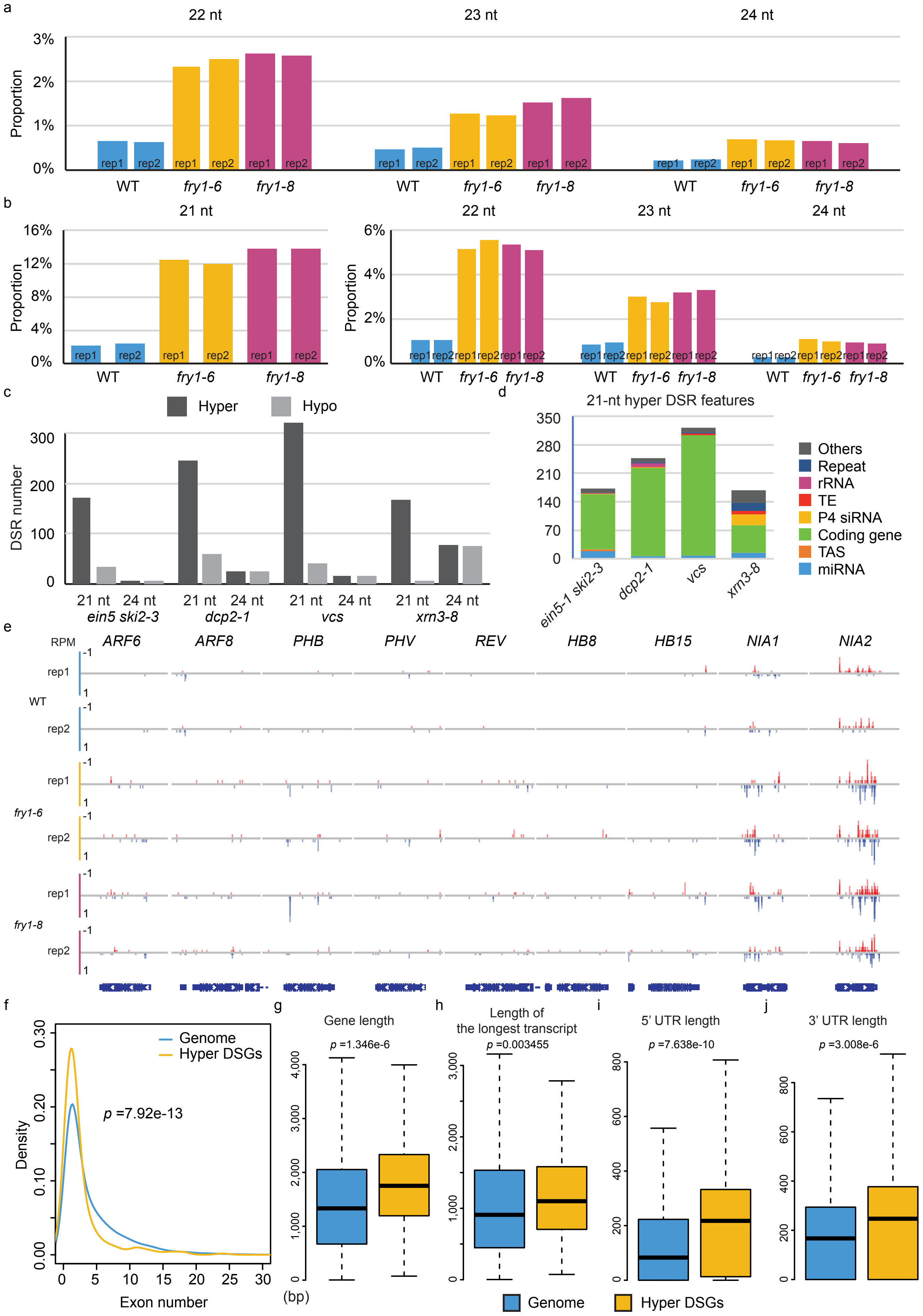

Supplementary Fig. 3 sRNAs from coding genes in various mutants

a, Proportion of 22-24 nt sRNAs from 21-nt hyper DSGs. The Y axis shows the proportion of sRNAs from combined 21-nt hyper DSGs (228) in both mutants in total mapped sRNA reads. **b**, Proportion of 21-24 nt sRNAs from 21-nt hyper DSGs. The Y axis shows the proportion of sRNAs from combined 21-nt hyper DSGs (228) in total mapped sRNA reads minus reads mapped to rRNA, tRNA, snoRNA, *etc.*. **c**, DSRs in published mutants. Only 21-nt and 24-nt DSRs are shown. There are more hyper DSRs in 21 nt compared to those in 24 nt in all mutants. **d**, Genomic classification of 21-nt hyper DSRs in the above-mentioned mutants. The annotation of DSRs was based on known genome features overlapping with the DSRs. Most 21-nt hyper DSRs in all mutants are from coding genes. There are also many hyper DSRs from P4 siRNA loci (from which siRNA biogenesis requires Pol IV), TE, and other repeats in *xrn3-8*, suggesting a different mechanism of sRNA biogenesis in *xrn3-8*. **e**, 21-nt sRNAs from miRNA targets and two non-targets mentioned in Zhang *et al.*, 2015[12]. There is a slightly increase in the levels of 21-nt sRNAs from both strands of the two positive controls *NIA1*/*2*, which are non-targets. However, there is no accumulation of 21-nt sRNAs from representative miRNA targets, including *ARF6*/*8*, *PHB*, *PHV*, *REV*, *HB8*/*15*, in *fry1* mutants. **f**, Genes with rogue 21-nt sRNAs in *fry1* have fewer exons. **g-j**, Genes with rogue 21-nt sRNAs in *fry1* tend to be longer than other genes in many aspects, including gene (**g**), longest transcript (**h**), 5’ UTR (**i**), and 3’ UTR (**j**). P values were calculated using the two-tailed Wilcoxon’s test. Source data are provided as a Source Data file.

Supplementary Figure 4

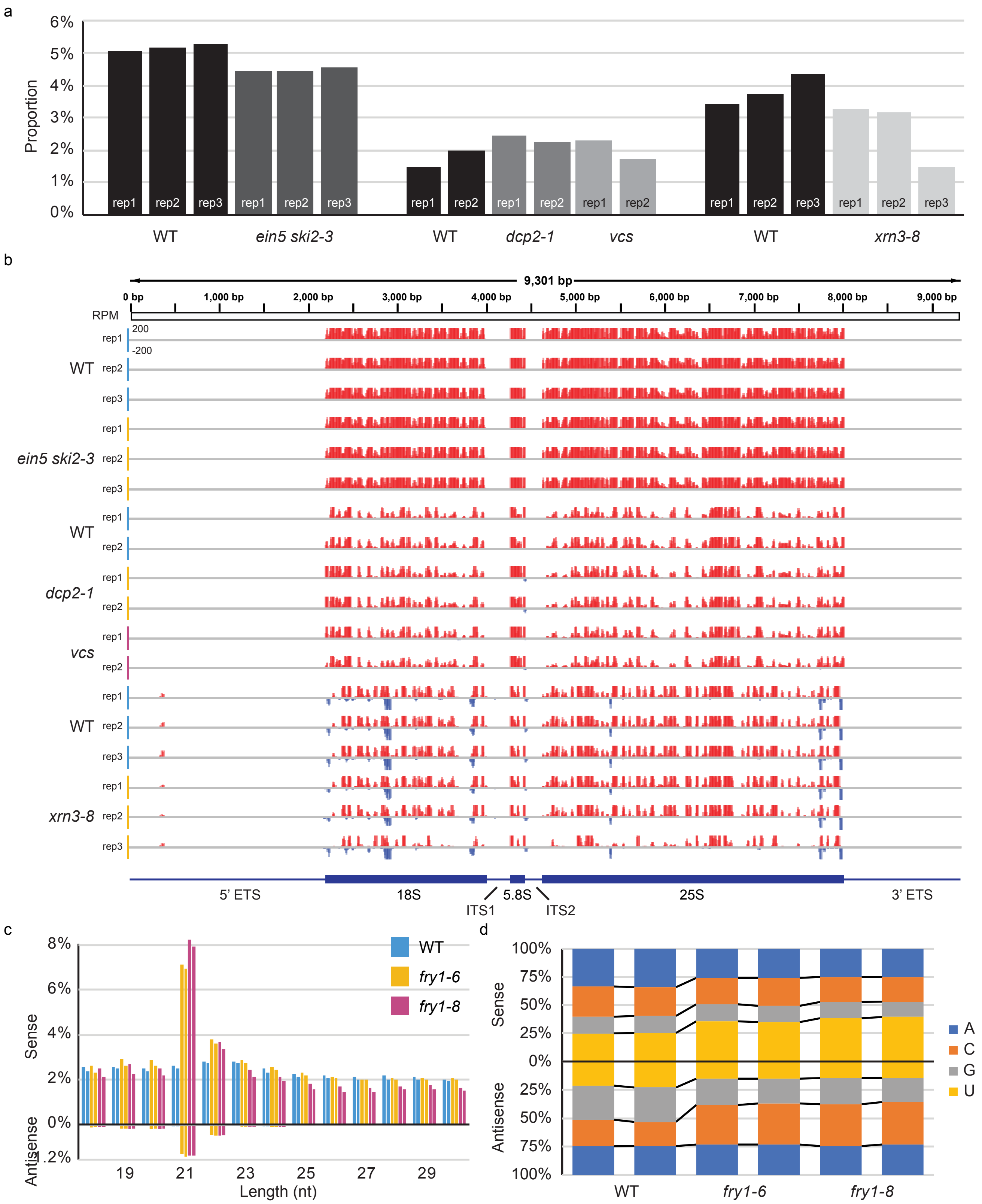

Supplementary Fig. 4 sRNAs from rDNA in various mutants

a, Proportion of 21-nt sRNAs from rDNA in 5'-3' RNA decay mutants. There is no enhanced accumulation of rRNA-derived sRNAs in these mutants compared to WT. Note that although rRNA fragments were removed after sequencing, they remained in the raw data of *dcp2-1* and *vcs*. The Y axis shows the proportion of sRNAs from rDNA in total mapped sRNA reads. **b**, IGV views for the chromosome 3 rDNA locus with 21-nt sRNAs in published datasets. There is no obvious siRNA accumulation along the rDNA in 5'-3' RNA decay mutants. The red and blue bars represent normalized read counts in RPM from the positive and negative strand, respectively. The rDNA locus is shown at the bottom. **c**, The length distribution of sRNA reads mapped to rDNA. Only 21-nt sRNAs accumulate drastically from both strands. The Y axis shows the proportion of sRNA from rDNA in total mapped sRNAs. **d**, The distribution of 5’ nucleotide identity of rDNA-derived sRNAs. The upper and lower parts represent the sense and antisense strands, respectively. 5’ U and 5’ C are preferred by the positive and negative strands, respectively. Source data are provided as a Source Data file.

Supplementary Figure 5

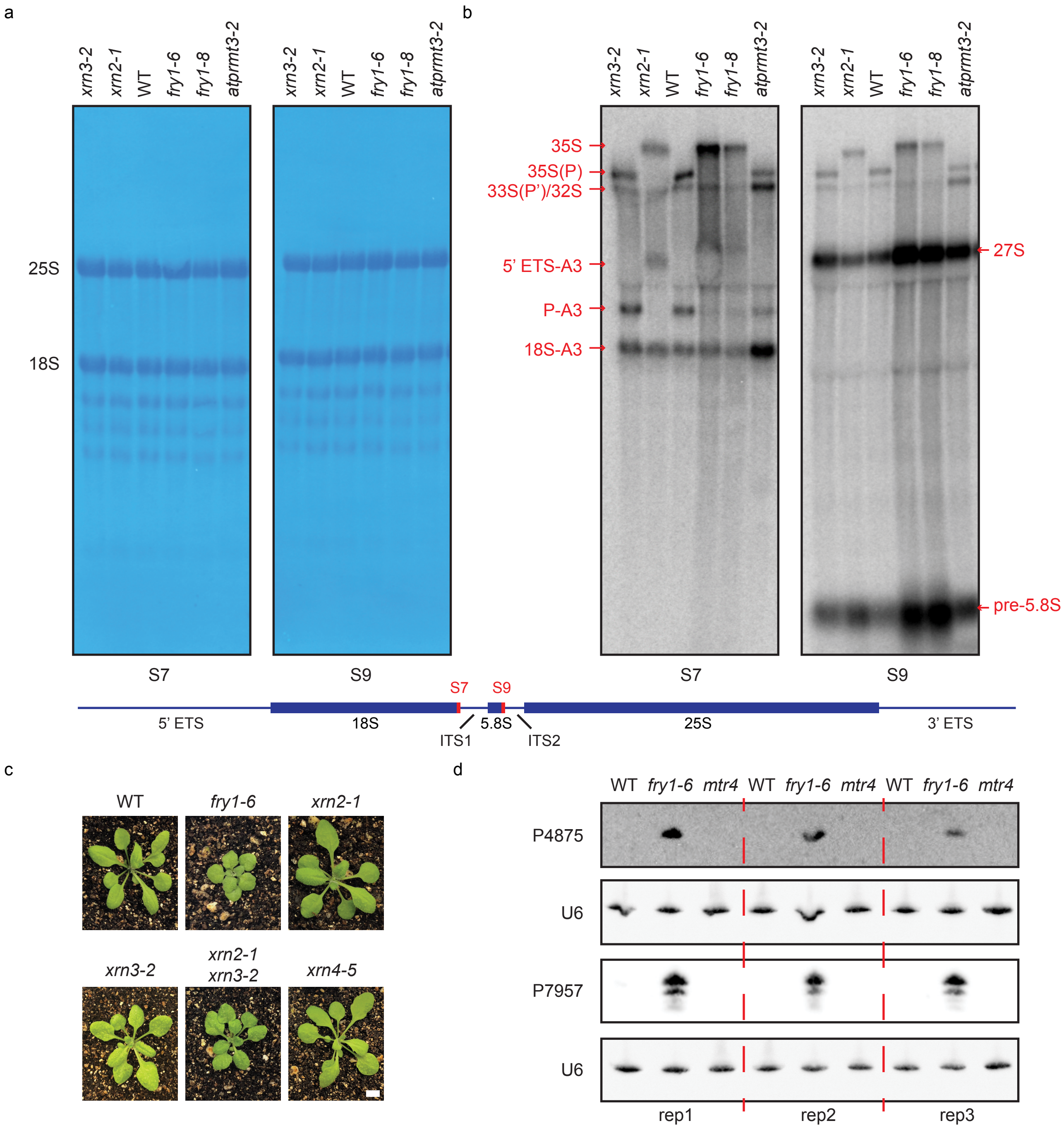

Supplementary Fig. 5 Correlation between rRNA-derived sRNA accumulation and defects in 5'-3' rRNA precursor processing

a, Stained RNA gels to show the abundance of mature rRNAs in WT and the indicated mutants. The abundance of mature rRNAs is not altered in *fry1* mutants. **b**, RNA gel blot assay to detect rRNA precursors using the S7 and S9 probes in various genotypes. *xrn2-1* and *atprmt3-2* serve as positive controls and *xrn3-2* serves as the negative control. The accumulation of abnormal rRNA precursors is greater in *fry1* than in the reported *xrn2-1* mutant. The rDNA locus with various features and the locations of probes are shown at the bottom. **c**, Plant phenotypes of *fry1* and *xrn* mutants. *fry1-6* resembles the *xrn2-1 xrn3-2* double mutant. **d**, RNA gel blot of rRNA-derived sRNAs in *mtr4*. There is no signal for rRNA-derived sRNAs in *mtr4* from three biological replicates. Source data are provided as a Source Data file.

Supplementary Figure 6

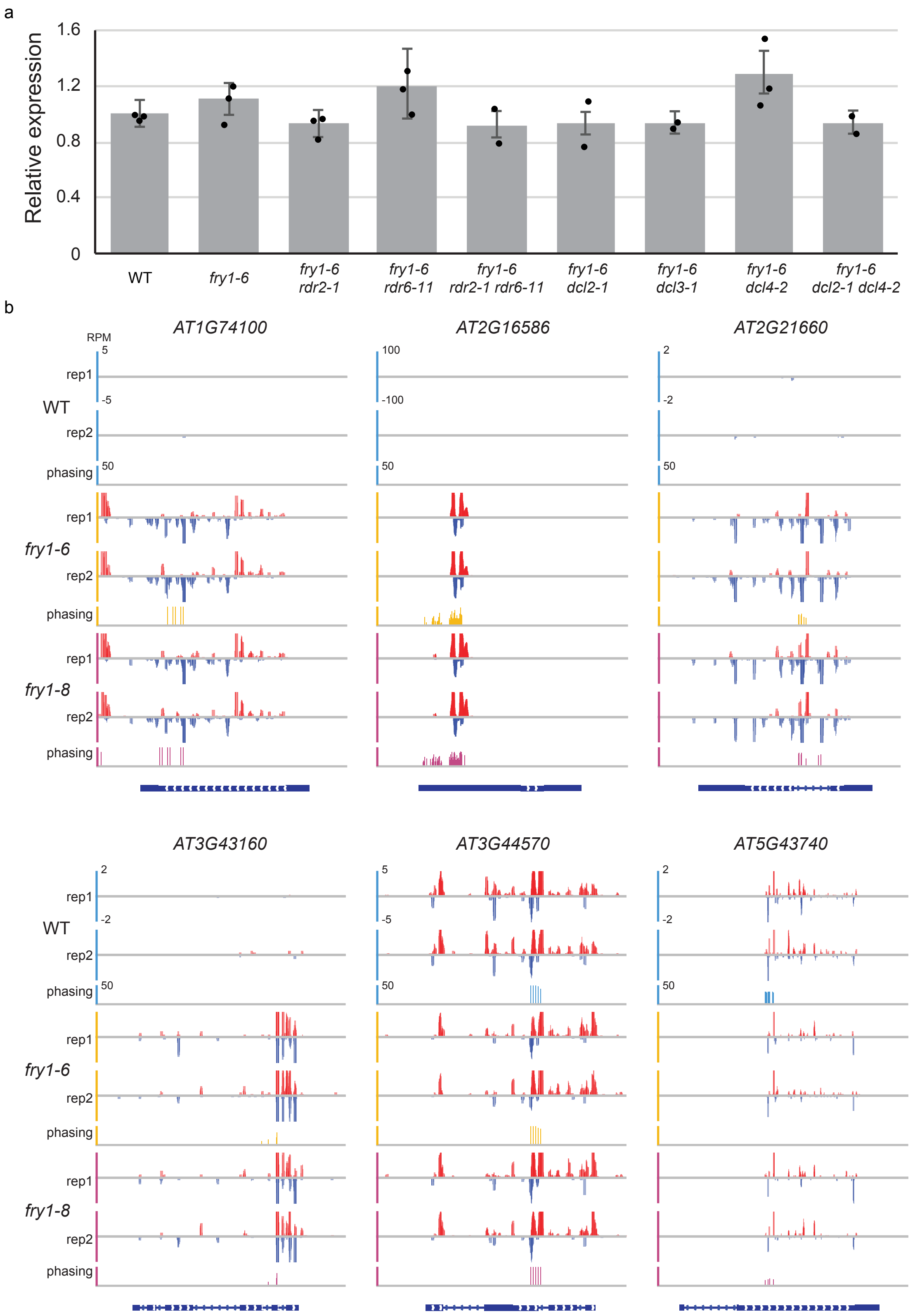

Supplementary Fig. 6 Phased siRNAs in WT and *fry1* mutants

a, The expression of *RDR1* in related mutants. There are no significant changes in the transcript levels of *RDR1* in all these mutants. Scale bars were defined by the SEM calculated from 2 or 3 biological replicates. P values were calculated by a two-tailed Student's t test. **b**, IGV views for four genes (AT1G74100, AT2G16586, AT2G21660, and AT3G43160) with phased, rogue 21-nt sRNAs in *fry1* and two genes (AT3G44570 and AT5G43740) known to generate phased siRNAs. The red and blue bars represent normalized read counts from the positive and negative strand, respectively. Gene structures are shown at the bottom. Source data are provided as a Source Data file.

Supplementary Figure 7

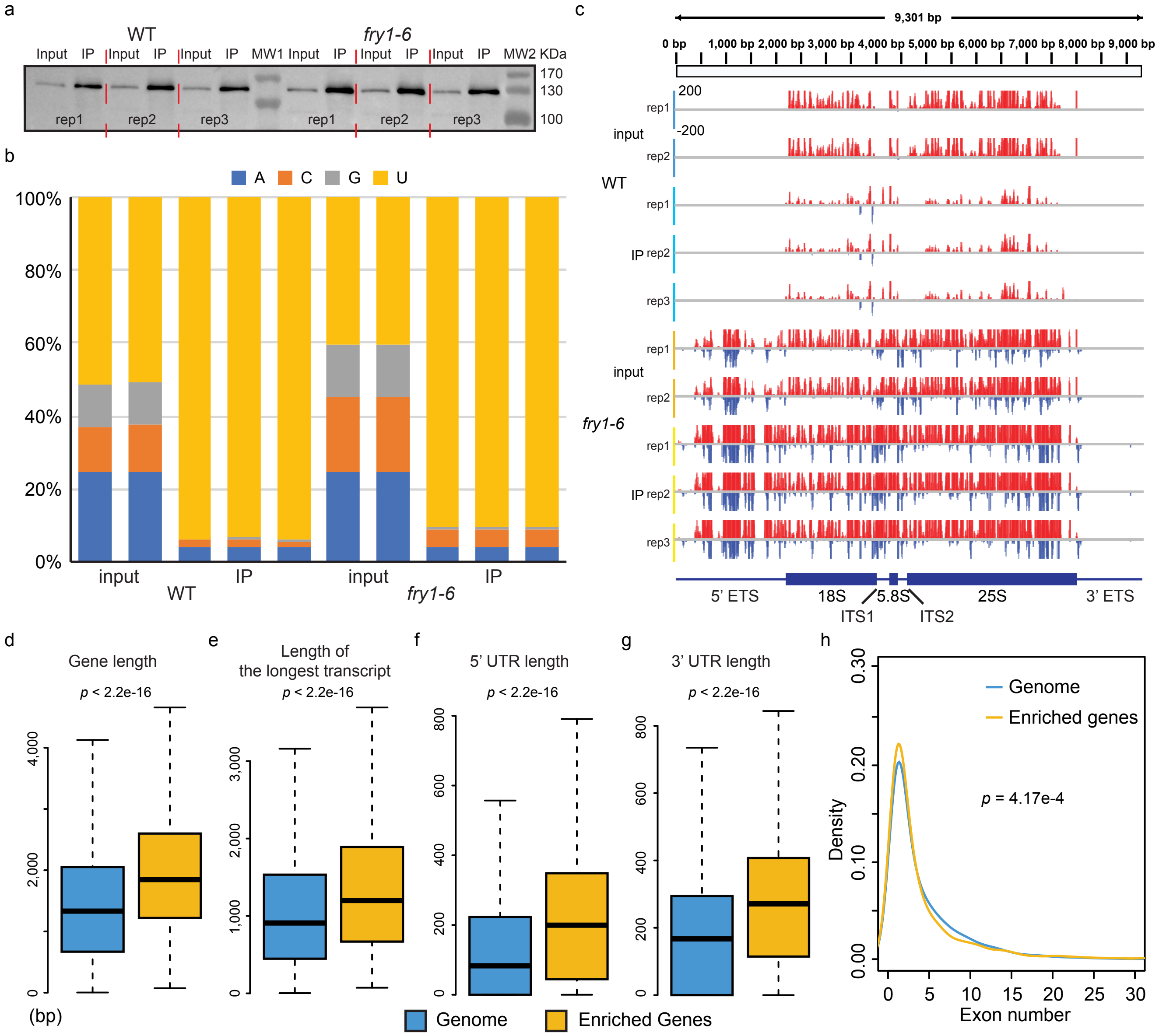

Supplementary Fig. 7 21-nt risiRNAs associate with AGO1

a, Detection of AGO1 by western blotting from input and immunoprecipitates (IP) in WT and *fry1-6*. Three biological replicates were conducted, and small RNAs were extracted from the IP samples for sRNA-seq. **b**, The distribution of 5' nucleotide identities of sRNAs associated with AGO1. 5' U is predominant in AGO1-associated sRNAs. **c**, IGV views of AGO1-associated 21-nt siRNAs from rDNA in WT and *fry1-6*. The red and blue bars represent normalized read counts in RPM from the positive and negative strand, respectively. The rDNA locus is shown at the bottom. **d-g**, Comparison between genes with enhanced 21-nt siRNA production in *fry1-6* and all annotated genes. These genes are longer in lengths of gene (**d**), transcript (**e**), 5' UTR (**f**), and 3' UTR (**g**). **h**, Genes with enhanced 21-nt siRNA production have fewer exons compared to all annotated genes in *Arabidopsis*. P values were calculated using the two-tailed Wilcoxon's test. Source data are provided as a Source Data file.

Supplementary Figure 8

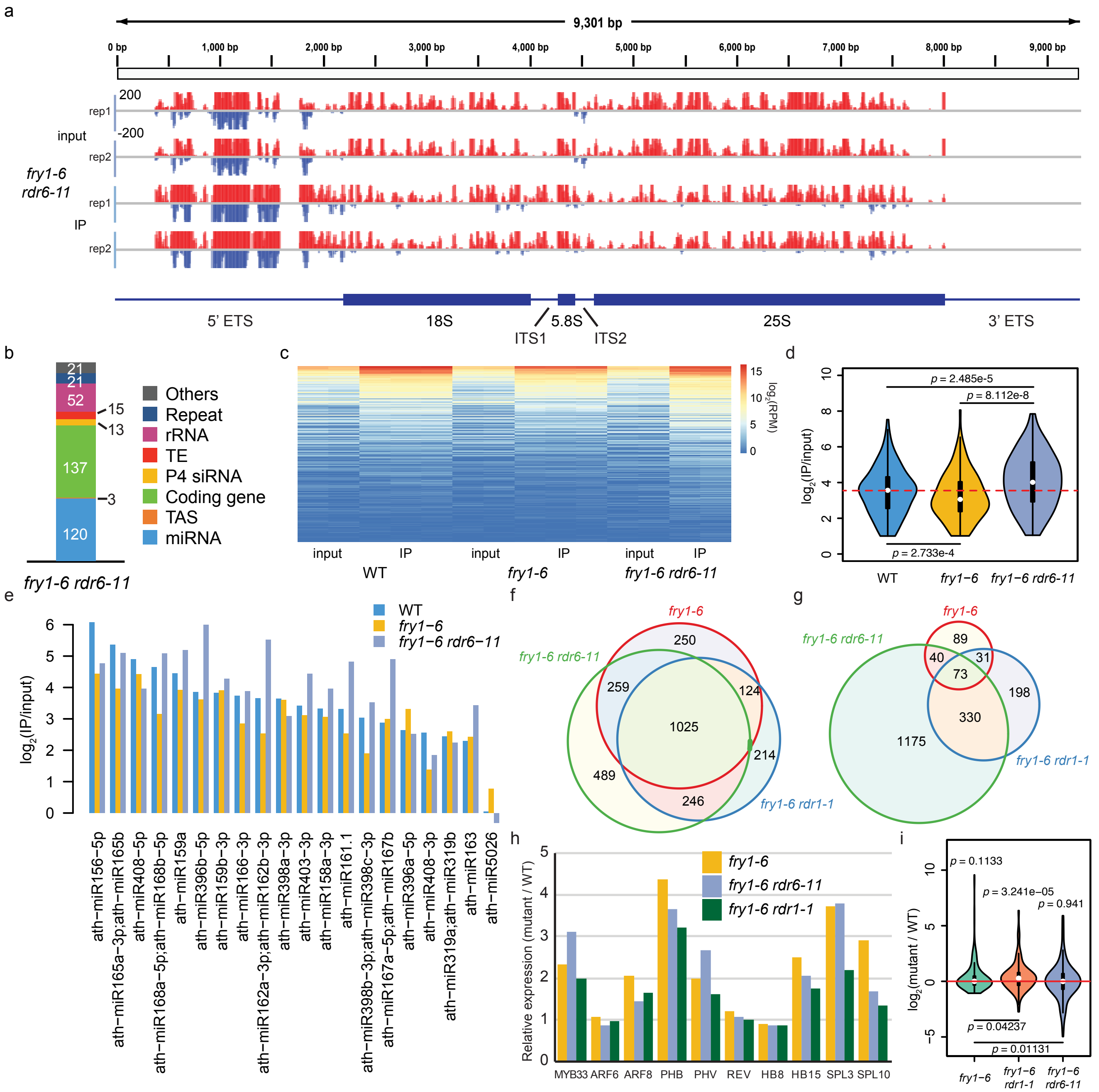

Supplementary Fig. 8 Partial removal of risiRNAs by *rdr6-11*

a, IGV views of total and AGO1-associated 21-nt siRNAs from rDNA in *fry1-6 rdr6-11*. The red and blue bars represent normalized read counts in RPM from the positive and negative strand, respectively. The rDNA locus is shown at the bottom. **b**, Genomic classification of 100-bp bins with an enrichment of 21-nt sRNAs in AGO1 IP vs. input from *fry1-6 rdr6-11*. Bins corresponding to *TAS* genes are barely present as a consequence of the *rdr6* mutation. The annotation was based on known genome features. **c**, A heatmap for all miRNAs in total sRNA sequencing and AGO1 IP-seq of WT, *fry1-6*, and *fry1-6 rdr6-11*. Each column represents a biological replicate, and each row represents a miRNA. The color shows the abundance of miRNAs in RPM. **d**, The efficiency of miRNA loading into AGO1 is reduced in *fry1-6* and restored by *rdr6-11* compared to WT. The miRNA loading efficiency is represented by the ratio of miRNA abundance in IP-seq and total sRNA sequencing. Only 121 miRNAs enriched in AGO1 IP (log2(IP/input) >= 1) were used for plotting. The red dashed line shows the median of miRNA loading efficiencies in WT. P values were calculated by a two-tailed paired Wilcoxon test. **e**, AGO1 loading efficiencies of the 20 most abundant miRNAs in WT; these miRNAs are also shown in Fig. 7b. **f**,**g**, Significantly up-regulated (**f**) and down-regulated (**g**) genes in *fry1-6*, *fry1-6 rdr6-11*, and *fry1-6 rdr1-1* as compared to WT. **h**, The relative expression of 10 representative of miRNA targets in *fry1-6*, *fry1-6 rdr6-11*, and *fry1-6 rdr1-1* as compared to WT. Seven of the 10 genes are expressed at a higher level in *fry1-6* (mutant/WT>1), and at least one of the *rdr6* and *rdr1* mutations can partially restore the expression levels. **i**, Expression levels of 186 genes generating 21-nt siRNAs in *fry1-6*;

the expression levels are from RNA-seq. The expression of these genes in *fry1-6 rdr1-1* is up-regulated significantly compared to that in WT, probably due to compromised siRNA biogenesis from the genes. Meanwhile, the expression is reduced in *fry1-6 rdr6-11* compared to that in *fry1-6*, in which siRNA biogenesis is enhanced. P values were calculated by a two-tailed paired Wilcoxon test. Source data are provided as a Source Data file.

Supplementary Figure 9

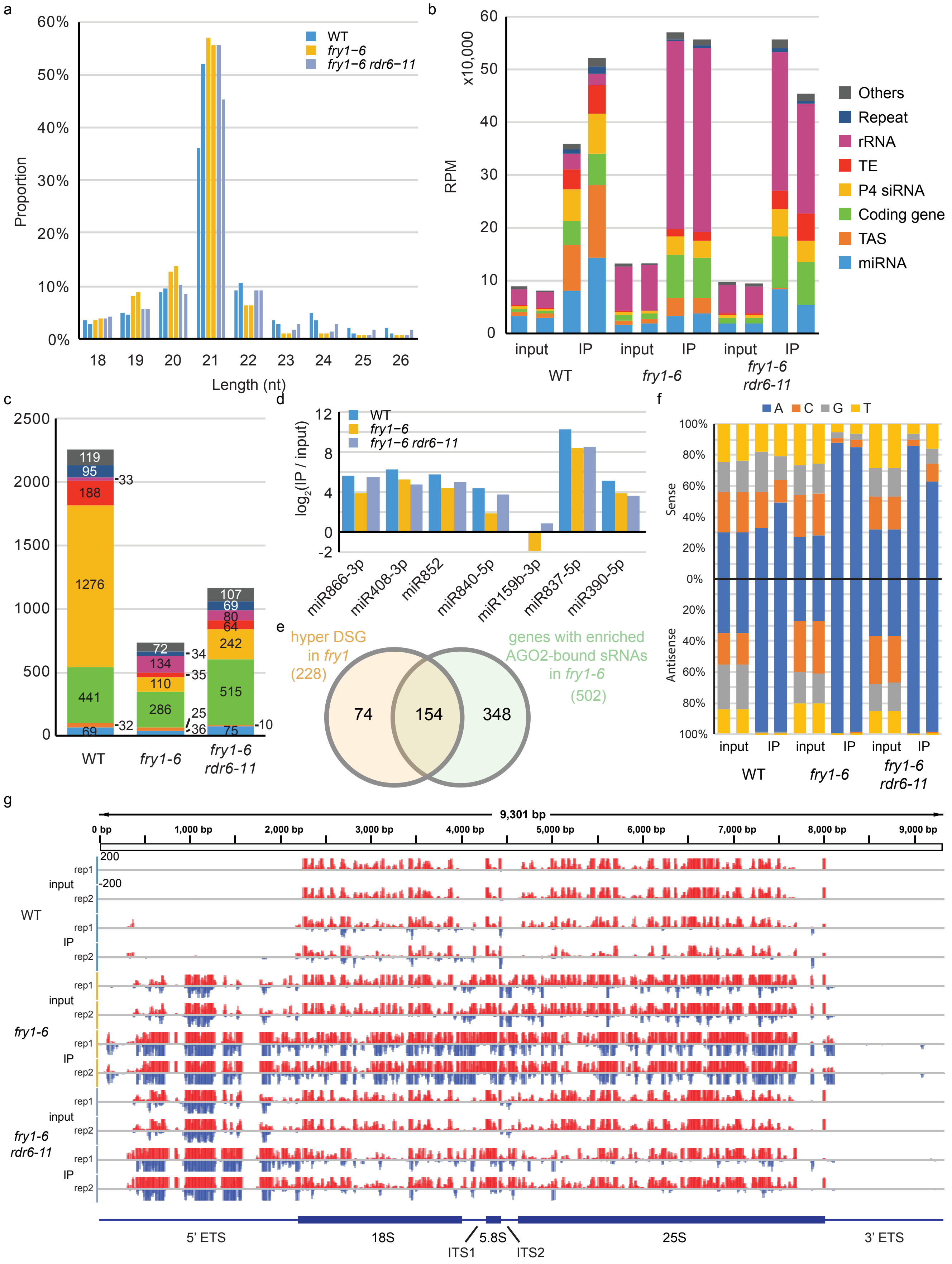

Supplementary Fig. 9 21-nt risiRNAs associate with AGO2

a, Length distribution of mapped reads from AGO2 IP in WT, *fry1-6*, and *fry1-6 rdr6-11*. **b**, Genomic classification of 21-nt AGO2-associated sRNAs. In WT, miRNAs and ta-siRNAs constitute a large portion of AGO2-associated 21-nt sRNAs. In *fry1-6*, similar to AGO1-associated 21-nt sRNAs, there is a drastic increase in rRNA-derived siRNAs. Also, the levels of AGO2-associated risiRNAs are reduced and those of miRNAs are partially restored in the *fry1-6 rdr6-11* double mutant. The Y axis shows the cumulative RPM values for sRNAs corresponding to different genomic features. **c**, Genomic classification of 100-bp bins with enhanced 21-nt sRNAs in AGO2 IP vs. input in WT, *fry1-6*, and *fry1-6 rdr6-11*. **d**, Loading efficiencies of known AGO2-bound miRNAs. **e**, Venn diagram for genes generating rogue 21-nt siRNAs. AGO2 binds to siRNAs from 154 of 228 genes generating siRNAs in *fry1-6*. **f**, The distribution of 5' nucleotide identities among sRNAs associated with AGO2. 5' A is predominant in AGO2-associated sRNAs. **g**, IGV views of total and AGO2-associated 21-nt siRNAs from rDNA in WT, *fry1-6*, and *fry1-6 rdr6-11*. The red and blue bars represent normalized read counts in RPM from the positive and negative strand, respectively. The rDNA locus is shown at the bottom. Source data are provided as a Source Data file.
