# Supplementary material for: FIERY1 promotes microRNA accumulation by suppressing rRNA-derived small interfering RNAs in Arabidopsis
Source: Nat Commun. 2019 Sep 27;10:4424. doi: 10.1038/s41467-019-12379-z (PMC6765019; doi:10.1038/s41467-019-12379-z)
Supplement: Supplementary file 3 — Description of Additional Supplementary Files [file 41467_2019_12379_MOESM3_ESM.pdf]

Description of Additional Supplementary Files

File Name: Supplementary Data 1
Description: Abundance of annotated miRNAs in fry1 and WT

File Name: Supplementary Data 2
Description: Hyper and hypo 21-nt DSRs in fry1

File Name: Supplementary Data 3
Description: Hyper and hypo 21-nt DSGs in fry1

File Name: Supplementary Data 4
Description: Overlap in genes with excess 21-nt siRNAs among different genotypes and statistical evaluation of significance of overlap

File Name: Supplementary Data 5
Description: Predicted targeting of DSGs by miRNAs

File Name: Supplementary Data 6
Description: Reads from sRNA-seq from immunoprecipitated AGO1 and AGO2 in fry1-6, fry1-6 rdr6-11, and WT

File Name: Supplementary Data 7
Description: Annotated miRNAs in input and AGO1 IP products from fry1-6, fry1-6 rdr6-11, and WT

File Name: Supplementary Data 8
Description: Annotated miRNAs in input and AGO2 IP products from fry1-6, fry1-6 rdr6-11, and WT

File Name: Supplementary Data 9
Description: Oligonucleotides used in this study
