# Supplementary material for: FIERY1 promotes microRNA accumulation by suppressing rRNA-derived small interfering RNAs in Arabidopsis
Source: Nat Commun. 2019 Sep 27;10:4424. doi: 10.1038/s41467-019-12379-z (PMC6765019; doi:10.1038/s41467-019-12379-z)
Supplement: Supplementary file 13 — Reporting Summary [file 41467_2019_12379_MOESM13_ESM.pdf]

nature research

Corresponding author(s): Jie Cui and Beixin Mo

Last updated by author(s): Aug 19, 2019

Reporting Summary

Nature Research wishes to improve the reproducibility of the work that we publish. This form provides structure for consistency and transparency in reporting. For further information on Nature Research policies, see Authors & Referees and the Editorial Policy Checklist .

Statistics

For all statistical analyses, confirm that the following items are present in the figure legend, table legend, main text, or Methods section.

n/a	Confirmed	
x		The exact sample size (*n*) for each experimental group/condition, given as a discrete number and unit of measurement
x		A statement on whether measurements were taken from distinct samples or whether the same sample was measured repeatedly
	x	The statistical test(s) used AND whether they are one- or two-sided<br>*Only common tests should be described solely by name; describe more complex techniques in the Methods section.*
x		A description of all covariates tested
x		A description of any assumptions or corrections, such as tests of normality and adjustment for multiple comparisons
	x	A full description of the statistical parameters including central tendency (e.g. means) or other basic estimates (e.g. regression coefficient) AND variation (e.g. standard deviation) or associated estimates of uncertainty (e.g. confidence intervals)
	x	For null hypothesis testing, the test statistic (e.g. *F*, *t*, *r*) with confidence intervals, effect sizes, degrees of freedom and *P* value noted<br>*Give P values as exact values whenever suitable.*
x		For Bayesian analysis, information on the choice of priors and Markov chain Monte Carlo settings
x		For hierarchical and complex designs, identification of the appropriate level for tests and full reporting of outcomes
x		Estimates of effect sizes (e.g. Cohen's *d*, Pearson's *r*), indicating how they were calculated

Our web collection on statistics for biologists contains articles on many of the points above.

Software and code

Policy information about availability of computer code

Data collection	All Illumina sRNA-seq data in this studies were collected by the manufacturer's software following the manufacturer's pipeline.
Data analysis	All sequencing data were analyzed by an in-house pipeline pRNASeqTools v0.6 [https://github.com/grubbybio/pRNASeqTools].

For manuscripts utilizing custom algorithms or software that are central to the research but not yet described in published literature, software must be made available to editors/reviewers. We strongly encourage code deposition in a community repository (e.g. GitHub). See the Nature Research guidelines for submitting code & software for further information.

Data

Policy information about availability of data

All manuscripts must include a data availability statement. This statement should provide the following information, where applicable:

- Accession codes, unique identifiers, or web links for publicly available datasets
- A list of figures that have associated raw data
- A description of any restrictions on data availability

The raw sequence data generated during this study were deposited into the NCBI GEO database under accession code GSE133461 [https://www.ncbi.nlm.nih.gov/geo/query/acc.cgi?acc=GSE133461]. The source data underlying Figs. 1b,c, 2a,c,d, 3a-c,e,g,h, 4b-e, 5a,b, 6a,c, 7a-e, and Supplementary Figs 1b-d,g, 2b-e, 3a-d,f-j, 4a,c,d, 5a-d, 6a, 7a,b,d-h, 8d,e,h,i, and 9a,b,d,f are provided as a Source Data file. The authors declare that any other supporting data is available from the corresponding author(s) upon request.

Field-specific reporting

Please select the one below that is the best fit for your research. If you are not sure, read the appropriate sections before making your selection.

x

October 2018

Life sciences study design

All studies must disclose on these points even when the disclosure is negative.

Sample size	No sample-size calculations were performed. Sample size was determined to be adequate based on the magnitude and consistency of measurements.
Data exclusions	No data were excluded in this study.
Replication	The reproducibility of biological replicates were shown by PCA of sRNA-seq datasets in Supplementary Figure 2.
Randomization	All experiments were in bulk, using RNA extracted from biological replicates which were consisted of around 20 seedlings of Arabidopsis thaliana.
Blinding	Investigators were not blinded to Arabidopsis genotypes during experiments. Data reported are not subjective.

Reporting for specific materials, systems and methods

We require information from authors about some types of materials, experimental systems and methods used in many studies. Here, indicate whether each material, system or method listed is relevant to your study. If you are not sure if a list item applies to your research, read the appropriate section before selecting a response.

Materials & experimental systems

n/a	Involved in the study	
☐	☒	Antibodies
☒	☐	Eukaryotic cell lines
☒	☐	Palaeontology
☒	☐	Animals and other organisms
☒	☐	Human research participants
☒	☐	Clinical data

Methods

n/a	Involved in the study	
☒	☐	ChIP-seq
☒	☐	Flow cytometry
☒	☐	MRI-based neuroimaging

Antibodies

Antibodies used	The rabbit polyclonal CTR1 antibody was prepared and purified by Shanghai ImmunoGen Biological Technology Co.,Ltd using the 1-485aa of CTR1 as the antigen. The rabbit polyclonal AGO1 antibody was purchased from Agrisera (Cat. AS09 527, Lot# 1711).
Validation	The validation of the CTR1 antibody was performed with WB by the provider using the antigen and by this study using the amiR-CTR1 transgenic plant (Supplementary Figure 1). The AGO1 antibody was validated by the manufacturer using WB in Arabidopsis [https://www.agrisera.com/en/artiklar/ago1-argonaute-1.html]
